# Treatment patterns and access among people with epilepsy of childbearing potential in Peru

**DOI:** 10.3389/fneur.2026.1916569

**Published:** 2026-08-11

**Authors:** Samantha E. Allen, Melissa T. Wardle, Luz M. Moyano, Percy Vilchez, Javier A. Bustos, Hector H. Garcia, Seth E. O’Neal

**Affiliations:** 1 Department of Neurology, University of California, Davis, Davis, CA, United States; 2 School of Public Health, Oregon Health and Science University – Portland State University, Portland, OR, United States; 3 School of Medicine, Universidad Nacional de Tumbes, Tumbes, Peru; 4 Centro de Salud Global, Universidad Peruana Cayetano Heredia, Tumbes, Peru; 5 Instituto Nacional de Ciencias Neurologicas, Lima, Peru; 6 Department of Microbiology, School of Sciences, Universidad Peruana Cayetano Heredia, Lima, Peru

**Keywords:** anti-seizure medications, epilepsy, global health, neurocysticercosis, reproductive health, resource-limited settings

## Abstract

**Objective:**

People with epilepsy of childbearing potential in low-resource settings face unique challenges related to anti-seizure medication (ASM) selection and access. These challenges may be particularly pronounced in regions where neurocysticercosis (NCC), a leading cause of acquired epilepsy worldwide, contributes substantially to the burden of disease. We characterized ASM prescribing patterns and factors associated with the use of higher-risk medications among people with epilepsy of childbearing potential in northern Peru.

**Methods:**

We analyzed data from females aged 15–49 years who were enrolled in a prospective, population-based epilepsy cohort in Tumbes, Peru, from 2006 to 2020. ASMs were categorized according to pregnancy-associated safety profiles as Lower-risk (lamotrigine, levetiracetam, oxcarbazepine, clonazepam, diazepam), Intermediate-High risk (carbamazepine, phenytoin, phenobarbital, topiramate), and Highest-risk (valproic acid). We evaluated ASM utilization, polytherapy, and factors associated with valproate use, the highest-risk medication.

**Results:**

Among 1,975 individuals with epilepsy, 685 were of childbearing potential. Approximately one-third met criteria for probable or definite NCC (34.9%). Nearly all participants (98.6%) were prescribed carbamazepine, phenytoin, phenobarbital, or valproic acid, while use of newer-generation agents was rare. Most prescriptions (86.3%) were classified as Intermediate-High-risk, and 12.8% as Highest-risk. In multivariable analyses, prior ASM use and polytherapy were independently associated with receipt of valproate.

**Discussion:**

In this population-based epilepsy cohort from northern Peru, ASM treatment among people of childbearing potential was overwhelmingly limited to older medications with an elevated risk of teratogenicity. These prescribing patterns likely reflect medication availability rather than clinical preference and highlight the challenges of implementing guideline-recommended epilepsy care in resource-constrained settings. Given the substantial burden of NCC-related epilepsy in this population, improving access to safer and more diverse ASM options may represent an important strategy for reducing treatment inequities among people with epilepsy of childbearing potential.

## 1 Introduction

Epilepsy affects an estimated 50 million people worldwide, with a disproportionate burden in low- and middle-income countries (LMICs) (1). In regions where *Taenia solium* is endemic, neurocysticercosis (NCC), an infection of the central nervous system caused by *T. solium*, is one of the leading causes of acquired epilepsy (2). For those afflicted, effective long-term seizure control remains a critical challenge, particularly in resource-limited settings where these infections are prevalent and access to anti-seizure medications (ASMs) is often constrained (3).

Among those impacted, people with epilepsy of childbearing potential (PWECP) represent a particularly vulnerable population that deserves special consideration. ASM choice in this group must balance seizure control with teratogenic risk and other potential adverse maternal and fetal outcomes. In determining ASM use in this population, guidelines recommend individualized therapy with preference for safer ASMs like levetiracetam and lamotrigine (4–6), together with preconception counseling; however, these safer ASMs are typically unavailable, and access to preconception services is limited in LMICs (5, 7). Undoubtedly, LMICs need guidelines that consider resource limitations. Unfortunately, the specificity of guidelines is limited by a paucity of data on ASM use and prescription patterns for PWECP in LMICs, particularly in populations with the further burden of NCC, where the burden of epilepsy may be higher and treatment gaps more pronounced (8).

Indeed, across LMICs, there is a dramatic epilepsy treatment gap in terms of access, diagnostic and therapeutic capabilities, quality of care, and other unmet health needs. Systematic reviews have estimated that more than 75% of individuals with epilepsy do not receive appropriate treatment, and access is especially poor in rural settings (8, 9). This is often due to limited availability of ASMs, high cost, inconsistent supply, and structural barriers in health systems (10).

In low-resource settings, prescription patterns are constrained by available ASMs and thus favor older, lower-cost drugs, such as phenobarbital, phenytoin, carbamazepine, and valproate, rather than newer agents with potentially better safety or tolerability profiles and lower risk of teratogenicity (10). In a recent systematic review in LMICs, the majority of studies (82.3%) reported use of first-generation ASMs, with limited adoption of newer medications (11, 12).

Northern Peru is highly endemic for *T. solium*, and a large, population-based cohort was established to study epilepsy in this region (13). This cohort has been previously leveraged to describe baseline ASM use, quality of life, and the epidemiology of NCC-associated epilepsy. However, ASM use among PWECP in this setting has not been systematically examined, representing a critical gap in understanding the intersection of epilepsy management, reproductive health, and NCC burden.

This study aims to characterize the patterns of ASM prescribing, including specific ASM risk profiles and polytherapy, and how these patterns vary by participant characteristics among PWECP in the Northern Peru epilepsy cohort. Understanding ASM use in this high-risk population has important implications for clinical management, reproductive counseling, and public health interventions in *T. solium*-endemic regions.

## 2 Methods

Study Design and Setting: Participants were drawn from a prospective, population-based epilepsy cohort established in Tumbes, Northern Peru, a region with high endemicity for *T. solium*. The cohort was designed to evaluate the epidemiology, clinical characteristics, and outcomes of epilepsy in people with and without NCC (13). Enrollment occurred between 2006 and 2020 through community-based screening in 107 villages, home visits, and self-referrals to local clinics. Participants underwent standardized clinical evaluations, including neurologic assessment, neuroimaging, serologic testing, and electroencephalography (EEG).

Study Population: Inclusion criteria for this analysis were: (1) diagnosis of epilepsy, defined as two or more unprovoked seizures separated by at least 24 h; (2) residence in a *T. solium*–endemic region; and (3) being of childbearing potential, defined as individuals identified as biologically female and aged 15–49 years. The age range of 15–49 years was selected in accordance with the World Health Organization (WHO) standard definition of reproductive age used for global reproductive health monitoring (14). Participants without epilepsy or outside the reproductive age range were excluded. Participants identified as male within the same reproductive age range (15–49 years) were included in a secondary analysis as a comparator group to contextualize ASM prescribing patterns in the overall population.

Data Collection: Demographic, clinical, and treatment information was collected using structured questionnaires administered by trained research personnel. ASM use was recorded, including type, dose, and use of concurrent medications at the time of baseline intake. ASM treatment decisions were tailored to individual patients’ circumstances and left to the treating physician’s discretion. ASM selection was not affected by the cohort study procedures. Additional data collected included year of recruitment, history of developmental delay, marital status, contraception use, seizure type, duration of epilepsy, and NCC diagnostic status using the modified Del Brutto criteria (15).

ASM Classification: ASMs were categorized by their pregnancy-associated risk of fetal malformations and adverse neurodevelopmental outcomes to define the primary outcome, ASM risk profile (16, 17). ASMs were classified as “Lower-risk” (lamotrigine, oxcarbazepine, clonazepam/diazepam, and levetiracetam), “Intermediate-High-risk” (carbamazepine, phenobarbital, phenytoin, and topiramate), and “Highest-risk” (valproic acid) (18–24). Given sparse observations in the lower-risk category (*n* = 6), these medications were excluded from the analysis comparing Intermediate-High and Highest-risk ASM prescriptions. Polytherapy was defined as the use of two or more ASMs concurrently. Participants receiving more than one ASM were assigned to the reproductive risk category corresponding to the highest-risk ASM in their regimen. ASMs considered reliably available in this region of Peru during the study period (2006 through 2020) include phenobarbital, phenytoin, carbamazepine, and valproic acid, all of which are on the national essential medicines list.

Statistical Analysis: Descriptive statistics are used to summarize demographic and clinical characteristics. Categorical variables are expressed as counts and percentages; continuous variables as means with standard deviation (SD). Prescription patterns were analyzed in relation to NCC status, seizure type, and participant demographic characteristics. Group comparisons were performed with Chi-square or Fisher’s exact tests for categorical variables and independent t-tests for continuous variables. Given the well-established and comparatively higher teratogenic risk associated with valproate, the primary analysis compared participants receiving valproate-containing regimens (“highest-risk”) with those receiving non-valproate anti-seizure medication regimens. The non-valproate group includes medications with heterogeneous reproductive safety profiles, including agents with established but more variable teratogenic risk. Multivariable logistic regression was used to evaluate factors associated with valproate or “Highest-risk” ASM use among those prescribed ASMs. ASM risk profile was used as the primary dependent variable, adjusting for age, marital status, epilepsy type among those with a known epilepsy classification (generalized or focal), use of ASM prior to enrollment, and number of ASMs prescribed. These variables were selected based on the results of the univariate analysis (*p* < 0.10) and known associates established in the literature (25, 26). Descriptive analyses were conducted using all available observations for each variable, with missing data reported where applicable. Regression statistical analyses, including multivariable \models, were performed using complete-case analysis and therefore included only participants with complete data for all variables included in the respective model. A significance threshold of *p* < 0.05 was used. Statistical analyses were performed using Stata version 19 (StataCorp, College Station, TX).

## 3 Results

Cohort Characteristics: Out of the full cohort of individuals with epilepsy totaling 1975 participants, there were a total of 685 biologically female individuals aged 15 to 49; this is the group defined as PWECP. Mean age was 30.1 years (SD 9.9). A little over half reported “married” as their marital status (52.6%). Data on contraception use had high missingness (66%), but among those surveyed, hormonal contraception (oral or injectable) was the most common, followed by no use of contraception. Long-acting, highly effective contraception (IUD or surgical) and condoms were infrequently reported. Approximately 10.5% had some history of developmental delay. Focal seizures predominated (60%) with a mean duration of diagnosis of 13.8 years (SD 11.5). A total of 239 participants (34.9%) met criteria for probable or definite NCC (Table 1), and the majority of cases had calcified lesions only (84%; data not shown).

**Table 1:** Baseline characteristics of cohort participants of childbearing potential.

Characteristic	No.* (%)
Participants aged 15–49	685
Average age (SD)	30.1 (9.9)
Married marital status	360 (52.6)
History of developmental delay	72 (10.5)
Method of contraception	
None	74 (10.8)
Tubal ligation/surgical	34 (5.0)
Oral Hormonal	33 (4.8)
Injectable Hormonal	71 (10.4)
Intrauterine Device	11 (1.6)
Condoms	11 (1.6)
Missing	451 (65.8)
Epilepsy Type	
Generalized	116 (16.9)
Focal	411 (60.0)
Unknown	12 (1.8)
Missing	146 (21.3)
NCC Diagnosis	239 (34.9)
Duration of epilepsy	
0–10 years	272 (39.7)
>10 years	310 (45.3)
Missing	103 (15.0)
ASM prior to recruitment	162 (23.6)
Number of ASMs prescribed at baseline	
None	138 (20.2)
Monotherapy	487 (71.1)
Polytherapy	60 (8.8)
ASM risk profile (*N* = 547)	
Lower Risk	5 (0.9)
Intermediate-High Risk	472 (86.3)
Highest Risk	70 (12.8)

*Proportions are presented out of 685 unless otherwise specified. Missingness observed for the following variables was marital status—3.6%, history of developmental delay—21.9%, and NCC diagnosis—9.1%.

**Figure 1:**
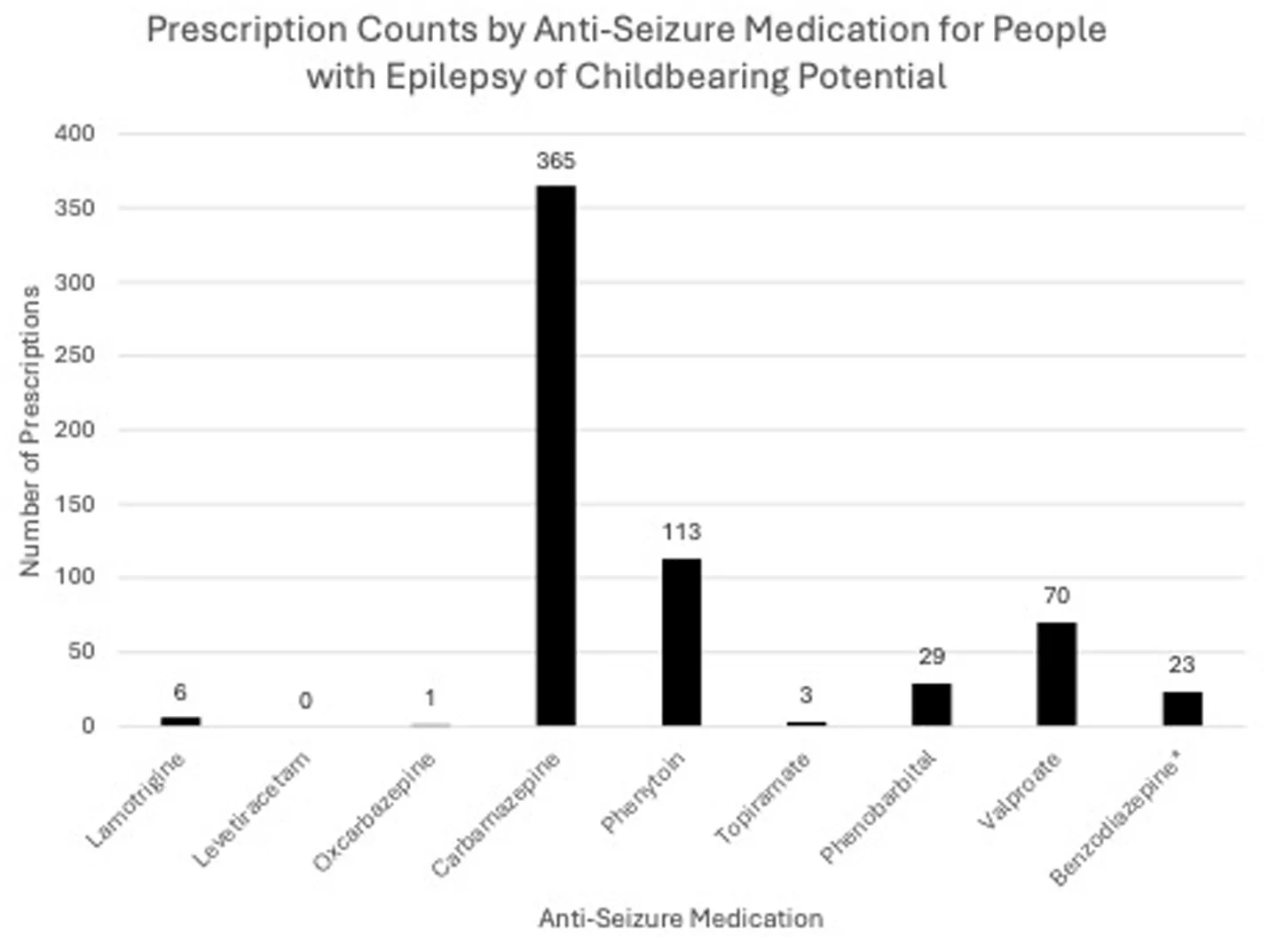
Anti-seizure medication prescription counts for cohort. *Benzodiazepine medications prescribed included clonazepam, diazepam, and clobazam. Participants receiving polytherapy contribute to multiple medication categories; therefore, the sum of counts exceeds the total number in the analytic population.

**Figure 2:**
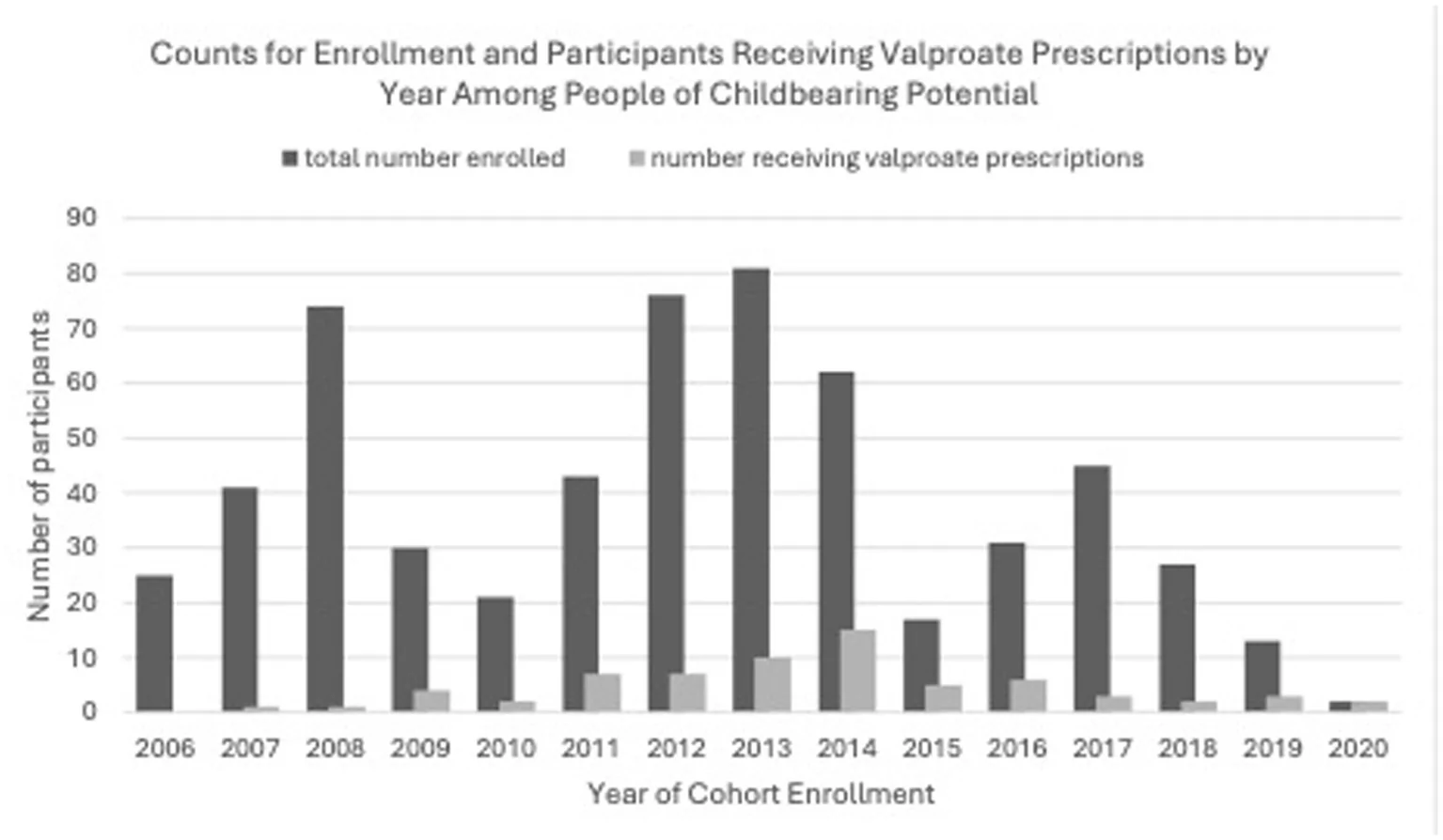
Number of valproate prescriptions by year of enrollment.

Anti-Seizure Medication Use: Among PWECP, approximately 80% (547/685) were prescribed at least one ASM at the time of baseline recruitment. Among those prescribed an ASM, monotherapy was used in 487 (89.0%) and polytherapy in 60 (11.0%). Carbamazepine was by far the most commonly prescribed ASM, followed by phenytoin and then valproate (Figure 1). Most baseline prescriptions fell into the “Intermediate-High risk” category (86%), with 12.8% receiving valproate, the medication in the “Highest-risk” category. ASM prescription patterns were evaluated by year of enrollment from 2006 to 2020. On average, approximately 40 participants (range: 2–81) were enrolled per year in this population, with peak enrollment occurring between 2012 and 2014. The percentage of participants receiving valproate was relatively stable over the sampled enrollment period, with a median of 12.3% in this population each year (Figure 2).

Factors Associated with Use of Highest-Risk ASM or Valproate Prescriptions: In the univariate model, marital status, ASM prescriptions before cohort enrollment, and the number of ASMs prescribed at the time of enrollment were all significantly associated with the ASM risk profile (Table 2). In the multivariable logistic regression model, Highest-risk ASM or valproate prescriptions were associated with a history of ASM prescription before cohort enrollment (OR 3.10, 95% CI 1.31, 7.37) and polytherapy (OR 3.82, 95% CI 1.59, 9.19) among participants prescribed Intermediate-High or Highest-risk ASMs after adjusting for age, marital status, and epilepsy type. No relationship was seen between marital status, age, and epilepsy type with ASM risk profile in the adjusted model (Table 3). Due to missing data, the logistic regression was limited to 209 participants. These 209 participants were more likely to have focal epilepsy, a shorter duration of epilepsy, have received an ASM prescription before recruitment, polytherapy prescription at baseline intake, and also more likely to be prescribed a Highest-risk category ASM when compared to those excluded (Supplementary Table S2).

**Table 2:** Factors associated with Valproate use, the highest risk anti-seizure medication.

Characteristic	Risk – n (%)		*p* value
	Intermediate-High*	Highest	
Participants aged 15–49	472	70	
Average age (SD)	29.7 (9.9)	27.7 (9.6)	0.10
Married marital status	246 (54.0)	27 (38.6)	**0.02**
History of developmental delay	47 (13.1)	9 (16.1)	0.54
Use of Contraception	111 (69.4)	10 (52.6)	0.14
Epilepsy Type			
Generalized	68 (18.4)	15 (23.1)	0.53
Focal	295 (79.7)	48 (73.9)	
Unknown	7 (1.9)	2 (3.1)	
NCC Diagnosis	172 (39.6)	23 (38.3)	0.85
Duration of epilepsy			
0–10 years	205 (50.5)	31 (50.0)	0.94
>10 years	201 (49.5)	31 (50.0)	
ASM prior to recruitment	103 (50.7)	35 (77.8)	**0.001**
No. of ASMs prescribed at baseline			
Monotherapy	435 (92.2)	47 (67.1)	**<0.001**
Polytherapy	37 (7.8)	23 (32.9)	

*Intermediate-high-risk medications include carbamazepine, phenytoin, phenobarbital, and topiramate. Values shown in bold are statistically significant at the *P* < 0.05 level.

**Table 3:** Associates of valproate use by multivariable logistic regression (*n* = 209).

Characteristic	Odds ratio	Standard error	*p*-value	Lower 95% CI	Upper 95% CI
Age					
	0.969	0.021	0.143	0.928	1.011
Marital Status					
Unmarried	1.00 (ref)				
Married	1.301	0.527	0.516	0.588	2.881
Epilepsy Classification					
Generalized	1.00 (ref)				
Focal	0.788	0.371	0.613	0.313	1.985
ASM prescribed prior to enrollment					
No	1.00 (ref)				
Yes	3.104	1.368	**0.010**	1.308	7.366
No. ASMs prescribed					
Monotherapy	1.00 (ref)				
Polytherapy	3.816	1.711	**0.003**	1.585	9.187

Male comparator population: Among biologically male participants, there were 637 in the reproductive age range of 15 to 49, with a mean age of 29 (SD 9.7). Males were less likely to be married than their female counterparts (*p* < 0.001) but had similar rates of developmental delay and distribution of epilepsy classification. For NCC, the distribution was also similar between females and male comparators in the same age range, with 38% of males meeting probable or definite NCC criteria. For prescriptions, 506/637 (79.4%) of males were prescribed at least one ASM. Monotherapy was used in 457/506 (90.3%) and polytherapy in 49/506 (9.7%). As with their female counterparts, most prescriptions were for medications in the “Intermediate-High Risk” category (87.9%), with nearly the entire remainder in the “Highest-risk” category (11.9%). Only one male participant in this age range was prescribed a “Lower-risk” regimen. ASM risk profiles did not differ significantly between sex groups (*p* > 0.05; Supplementary Table S1).

## 4 Discussion

In this large, population-based epilepsy cohort from a *Taenia solium*–endemic region of Northern Peru, we observed that during the study period from 2006 to 2020, PWECP were predominantly treated with carbamazepine, phenytoin, phenobarbital, and valproate, the ASMs reliably available in this region during this time. While phenytoin, phenobarbital, and carbamazepine are categorized as “Intermediate-High-risk,” valproate is “Highest-risk” for fetal malformations and adverse neurodevelopmental outcomes (26–29). Despite evidence supporting the use of agents such as lamotrigine and levetiracetam for PWECP, the prescribing patterns observed in our cohort reflect local formulary constraints. This is consistent with prior work in LMICs demonstrating that older-generation ASMs remain the backbone of epilepsy care, with newer, safer agents rarely accessible through public health systems (10, 12, 30, 31). Our data extend these observations to a uniquely vulnerable group, PWECP living in a *T. solium*-endemic region, where both seizure burden and reproductive risk are elevated.

Prenatal exposure to valproate is associated with a substantially increased risk of major congenital malformations, including neural tube defects, craniofacial anomalies, and cardiac malformations, with risk rising in a dose-dependent manner. In addition to structural defects, in utero valproate exposure is linked to long-term neurodevelopmental impairments, including reduced cognitive performance, language delays, and increased risk of autism spectrum disorder and attention-deficit/hyperactivity disorder, also in a dose-dependent manner (4, 27, 28, 32). In this cohort, while there is a clear preference for avoiding valproate where possible, more than 12% of PWECP are still receiving it. The similarity in ASM prescription risk profiles between PWECP and males in the same age range suggests that reproductive status does not meaningfully influence prescribing decisions in this setting.

The social implications and financial cost of valproate use in pregnancy are staggering. The risk of major congenital malformations alone, the most overt risk, for all doses of valproate is over 9%. For doses of valproate 1,450 mg and over, the risk of major congenital malformation is over 25% (24). The cost and visibility of adverse neurodevelopmental outcomes in children of people taking valproate in pregnancy are less clearly seen, but no less impactful. From a financial perspective alone, it is estimated in the United States that increased costs of special education and other medical needs for children with neurodevelopmental deficits attributable to in utero ASM exposure total approximately 626 million dollars each year (33). This estimate does not include the costs of malformations and behavioral disorders (e.g., autism) nor the emotional and financial impact on the child’s family.

In our analysis, we identified ASM prescriptions before enrollment and polytherapy at intake as predictors of valproate use. The association with prior ASM prescription before enrollment and polytherapy is likely reflective of greater seizure severity or drug resistance. Unfortunately, polytherapy, regardless of valproate inclusion in the ASM regimen, in itself has been identified as an independent risk factor for adverse fetal neurodevelopmental outcomes (34, 35).

Despite the clear clinical importance of balancing seizure control with reproductive risk, there is limited guidance tailored to low-resource settings where newer-generation ASMs like lamotrigine and levetiracetam are not readily available. Most guidelines are developed based on practice contexts where a range of newer ASMs can be accessed and monitored with reliable laboratory support (4, 5). In contrast, in many LMICs, these newer agents are either unavailable or unaffordable for most (10, 30).

This lack of context-specific guidance for LMICs leaves clinicians and patients navigating highly complex decisions with limited supporting evidence when choosing among available medications that carry greater teratogenic potential. There is currently no widely accepted, resource-stratified framework to support ASM selection in these circumstances, nor sufficient data on outcomes among PWECP treated with first-generation ASMs under real-world constraints.

While current guidelines from high income country major health policy leaders including the America Academy of Neurology (AAN), US Food and Drug Administration (FDA) and European Medicines Agency (EMA) make clear the importance of avoiding valproate in PWECP (36–38), even the WHO with its more global, and broadly inclusive mission still falls short of providing recommendations beyond this clear prohibition (39). The absence of guidance is likely due mostly to a scarcity of longitudinal, large-scale research in LMICs that examines ASM effectiveness, safety, and reproductive outcomes when newer ASMs are not available. As a result, clinicians frequently must rely on best clinical judgment, balancing seizure type, severity, and potential reproductive risk within the narrow range of medications available.

These findings highlight the need for pragmatic, context-aware guidance to support ASM selection when choices are inherently limited. Such guidance would explicitly address scenarios in which newer-generation ASMs are unavailable and offer risk-mitigation strategies, including reproductive health counseling. Strengthening clinical support tools, including simplified decision aids that incorporate medication availability, seizure type, teratogenic risk, and reproductive intentions, could help standardize care and improve shared decision-making.

Strengths of this study include its population-based design, large sample size, and systematic clinical characterization, including NCC status. However, several limitations should be acknowledged. We lack data on participants’ family planning or on pregnancy intentions. We also lack data on specific individualized provider decision-making parameters. There is also significant missingness among numerous covariates, which particularly skews the representativeness of the sub-population used in the multivariate regression analysis, limiting interpretability (Supplementary Table S2). Nonetheless, missingness is inherent to medical record data when leveraged as a secondary data source. Despite these limitations, these data are important to present given the scarcity of information capturing the experience of this epilepsy population.

Finally, this data is from 2006 to 2020, and there have been changes to ASM availability in Peru since this time. Notably, levetiracetam was evaluated for inclusion in Peru’s National Formulary in 2017 and incorporated within a complementary list for refractory epilepsy. In the most recent 2023 formulary, it remains a restricted therapy requiring specialist justification and institutional approval (40). With this update, levetiracetam is now more widely available in Peru, but due to restrictions, its availability in rural settings remains limited, where access to specialty care and approval pathways is constrained. In rural Peru, treatment continues to include predominantly older ASMs as first-line options.

In summary, this study reveals that in a *T. solium* endemic, resource-limited setting, ASM prescribing for PWECP is more likely shaped by medication availability and seizure control needs, rather than teratogenic risk profiles. Valproate and other older ASMs are widely used not because clinicians are unaware of reproductive risks, but because these agents are often the most effective and reliable treatment options available. This underscores the critical need for guidance that recognizes the realities of low-resource epilepsy care and supports clinicians and patients in making informed decisions that best balance seizure control with reproductive considerations.

## Data Availability

The raw data supporting the conclusions of this article will be made available by the authors, without undue reservation.
