# Supplementary material for: Treatment patterns and access among people with epilepsy of childbearing potential in Peru
Source: Front Neurol. 2026 Aug 11;17:1916569. doi: 10.3389/fneur.2026.1916569 (PMC13503225; doi:10.3389/fneur.2026.1916569)
Supplement: Supplementary file 1 [file Data_Sheet_1.PDF]

Supplemental Materials

Table S1: Baseline characteristics of Male and Female Participants

	**Females No. (%)**	**Males No. (%)**	**P value**
**Participants aged 15-49**	685	637	
**Average age (SD)**	30.1 (9.9)	29.1 (9.7)	0.11
**Married marital status**	360 (52.6)	226 (37.1)	**<0.001**
**History of developmental delay**	72 (10.5)	73 (11.5)	0.48
**Epilepsy Type**			
Generalized	116 (16.9)	115 (18.1)	0.60
Focal	411 (60.0)	359 (56.4)	
Unknown	12 (1.8)	12 (1.9)	
Missing	146 (21.3)	151 (23.7)	
**NCC Diagnosis**	239 (34.9)	243 (38.2)	0.26
**Duration of epilepsy**			
0-10 years	272 (39.7)	273 (42.9)	0.48
>10 years	310 (45.3)	277 (43.5)	
Missing	103 (15.0)	87 (13.7)	
**ASM prior to recruitment**	162 (23.6)	158 (24.8)	0.62
**Number of ASMs prescribed at baseline**			
None	138 (20.2)	131 (20.6)	0.78
Monotherapy	487 (71.1)	457 (71.7)	
Polytherapy	60 (8.8)	49 (7.7)	
**ASM risk profile***			
Lower Risk	5 (0.9)	1 (0.2)	0.27
Intermediate-High Risk	472 (86.3)	445 (87.9)	
Highest Risk	70 (12.8)	60 (11.9)	

* Denominator out of total participants prescribed ASMs at baseline (n=547 for females and n=506 for males)

Table S2: Characteristics of Participants Included in the Regression Analysis (n=209) compared with those excluded due to missingness (n=338)

	Included n (%)	Excluded n (%)	P value
**Participants aged 15-49**	209	338	
**Average age (SD)**	29.2 (9.8)	29.7 (9.9)	0.53
**Married marital status**	116 (55.5)	160 (49.7)	0.19
**History of developmental delay**	20 (12.7)	38 (14.5)	0.62
**Epilepsy Type**			
Generalized	38 (18.2)	47 (20.5)	**0.01**
Focal	171 (81.8)	173 (75.6)	
Unknown	0 (0)	9 (3.9)	
**NCC Diagnosis**	75 (38.7)	121 (39.8)	0.80
**Duration of epilepsy**			
0-10 years	112 (57.7)	125 (45.0)	**0.01**
>10 years	82 (42.3)	153 (55.0)	
**ASM prior to recruitment**	111 (53.1)	28 (8.3)	**<0.001**
**Number of ASMs prescribed at baseline**			
Monotherapy	178 (85.2)	309 (91.4)	**0.02**
Polytherapy	31 (14.8)	29 (8.6)	
**ASM risk profile**			
Lower Risk	0 (0)	5 (1.5)	**<0.001**
Intermediate-High Risk	168 (80.4)	304 (89.9)	
Highest Risk	41 (19.6)	29 (8.6)	
